# Intestinal Metabolites Influence Macrophage Phagocytosis and Clearance of Bacterial Infection

**DOI:** 10.3389/fcimb.2021.622491

**Published:** 2021-07-19

**Authors:** Amy A. O’Callaghan, Elaine Dempsey, Namrata Iyer, Sarah Stiegeler, Kevin Mercurio, Sinéad C. Corr

**Affiliations:** ^1^ Department of Microbiology, Moyne Institute of Preventative Medicine, School of Genetics and Microbiology, Trinity College Dublin, Dublin, Ireland; ^2^ APC Microbiome Ireland, University College Cork, Cork, Ireland

**Keywords:** metabolite, succinate, lactate, itaconate, macrophage, intestinal infection

## Abstract

The metabolite-rich environment that is the intestinal lumen contains metabolic by-products deriving from microbial fermentation and host cell metabolism, with resident macrophages being constantly exposed to this metabolic flux. Succinate, lactate and itaconate are three metabolites secreted by primed macrophages due to a fragmented tri-carboxylic acid (TCA) cycle. Additionally, succinate and lactate are known by-products of microbial fermentation. How these metabolites impact biological functioning of resident macrophages particularly in response to bacterial infection remains poorly understood. We have investigated the potential influence of these metabolites on macrophage phagocytosis and clearance of *Escherichia coli* (*E. coli*) infection. Treatment of murine bone-marrow-derived macrophages (BMDMs) with succinate reduced numbers of intracellular *E. coli* early during infection, while lactate-treated BMDMs displayed no difference throughout the course of infection. Treatment of BMDMs with itaconate lead to higher levels of intracellular *E. coli* early in the infection with bacterial burden subsequently reduced at later time-points compared to untreated macrophages, indicative of enhanced engulfment and killing capabilities of macrophages in response to itaconate. Expression of engulfment mediators MARCKS, RhoB, and CDC42 were reduced or unchanged following succinate or lactate treatment and increased in itaconate-treated macrophages following *E. coli* infection. Nitric oxide (NO) levels varied while pro- and anti-inflammatory cytokines differed in secretory levels in all metabolite-treated macrophages post-infection with *E. coli* or in response to lipopolysaccharide (LPS) stimulation. Finally, the basal phenotypic profile of metabolite-treated macrophages was altered according to marker gene expression, describing how fluid macrophage phenotype can be in response to the microenvironment. Collectively, our data suggests that microbe- and host-derived metabolites can drive distinct macrophage functional phenotypes in response to infection, whereby succinate and itaconate regulate phagocytosis and bactericidal mechanisms, limiting the intracellular bacterial niche and impeding the pathogenesis of infection.

## Introduction

The gastrointestinal lumen is a metabolite rich environment with particular metabolites now known to derive from primed host-immune cells as well as the gut microbiota (Jha et al., 2015; Maslowski, 2019). Macrophages display a ‘broken’ Krebs cycle or TCA cycle in response to the bacterial cell wall component LPS, such that in M1 pro-inflammatory macrophages, it is not a cycle that occurs but instead a fragmented process, resulting in the secretion of large volumes of metabolites such as succinate, lactate and itaconate (Jha et al., 2015), with these distinct metabolites present at high concentrations in an inflamed intestine. Reprogramming of this specific metabolic pathway upon activation is also for the production of metabolites that can act as immune signaling molecules, such as citrate, which has been linked to macrophage activation and is involved in the production of the important metabolite itaconate (Williams and O’Neill, 2018). Although there is growing evidence that metabolites are associated with distinct macrophage phenotypes, knowledge of their role in inducing a specific phenotype based on functional readouts is limited.

Macrophages are versatile cells in the immune system that provide protection against a wide variety of infections. Besides the most characteristic tasks as phagocytic killers and antigen-presenting cells, macrophages are essential in the control of inflammatory responses and in the repair of damaged tissues. Macrophages can express different functional programs in response to environmental signals to adapt their function to the needs of a particular immunological situation. Depending on the microenvironment, macrophages have been classified as classically activated M1 or alternatively activated M2. M1 macrophages are characterized by a high microbicidal capacity through reactive oxygen and nitrogen species and secretion of pro-inflammatory cytokines, while M2 macrophages produce high levels of anti-inflammatory cytokines and express cell surface markers that participate in tissue remodeling and resolution of inflammation (Saha et al., 2017). Complexity of function and metabolic rewiring means it is necessary to understand the phenotypic variation that can occur in response to specific stimuli, gut metabolites for example, to perhaps reveal ways in which macrophages may be altered or influenced in the face of intestinal inflammation or infection.

Here, we have investigated the ability of three gut associated metabolites succinate, lactate and itaconate to influence the functional response of macrophages to infection. We show that succinate limits the ability of macrophage to engulf *E. coli* while itaconate boosts phagocytic and bactericidal capability, thereby reducing the intracellular niche of this pathogen, and promoting clearance of the infection.

## Materials And Methods

### Bacterial Strains and Growth Conditions

Pathogenic *E. coli* NCTC12900 (shiga toxin negative O157:H7) was grown in Luria Bertani (LB) broth at 37°C shaking and plated onto LB agar. All experiments performed using *E. coli* NCTC12900 were performed in ClassII Biohazard facilities.

### Animals

C57BL/6J mice were maintained in ventilated cages at 21 ± 1°C, humidity 50 ± 10% and with a 12h-light/12h-dark light cycle under specific pathogen-free conditions, in line with Irish and European Union regulations. Food and water were monitored and available *ad libitum* throughout the experiments. All experiments involving use of mice or mouse tissue were subject to ethical approval by the Animal Research Ethics Committee (AREC), a Level 2 ethics committee responsible for reviewing the proposed use of animals in teaching and research at Trinity College Dublin, and were carried out in accordance with the recommendations of the Irish Health Products Regulatory Authority, the competent authority responsible for the implementation of Directive 2010/63/EU on the protection of animals used for scientific purposes in accordance with the requirements of the S.I No 543 of 2012.

### Isolation of BMDMs

Tibia and femur from 6 to 8 week-old C57BL/6J mice were collected in ice cold phosphate buffered saline (PBS). Bones were sterilized with 70% ethanol, cleaned and flushed with a 25-G needle using cold Dulbecco’s modified eagle medium (DMEM) (Gibco) supplemented with 10% fetal calf serum (FCS) and 1% penicillin-streptomycin (Sigma Aldrich). Following red-blood cell lysis, cells were seeded onto non-cell culture coated 10 cm dishes in complete DMEM containing 20% macrophage-colony stimulating factor (M-CSF) containing L929 media and incubated 37°C, with 5% CO2 for 6 days. Subsequently, BMDMs were seeded at 5 x 10^5^ cells/ml in 12 and 24-well tissue culture plates (Starstedt) in DMEM containing 10% L929 and 10% FCS. Macrophages derived using this method are considered M0 (resting phase/steady state/unpolarized macrophages) (Ying et al., 2013).

### Macrophage Treatments

BMDMs were treated with metabolites diethyl succinate at 2.5 mM (Sigma), sodium DL-lactate at 75 mM (Sigma) or dimethyl itaconate at 250 μM (Fluorochem) for 3 hours (hrs). A 3-hour pre-treatment was adapted from Mills et al. to enable metabolites to induce intracellular changes (Mills et al., 2018) while following a dose-dependent assay (**Supplementary Figure 1**). The dose-dependent concentrations were chosen based on physiological and pathological levels of succinate, lactate and itaconate that have been previously reported (Cordes et al., 2016; Errea et al., 2016; Mills et al., 2018). The following concentrations were identified for subsequent experiments: diethyl succinate 2.5 mM, sodium DL-lactate 75 mM,dimethyl itaconate 250 μM. BMDMs were treated with distinct concentrations of metabolites for 3 hours followed by bacterial lipopolysaccharide (LPS; Enzo) at 100 µg/ml for 24 hours.

### Phagocytosis Assay

Following metabolite pre-treatment of BMDMs, *E. coli* NCTC12900 was added at a multiplicity of infection (MOI) 20:1 for 15 minutes, and media subsequently replaced with DMEM containing gentamicin (100µg/ml). At various timepoints, monolayers were washed and lysed and subsequently plated on to LB agar plates for determination of rates of phagocytosis, expressed as Log CFU/ml. Gentamicin concentration was lowered to 10µg/ml for later time-points.

### Cell Viability Assay

For viability assay, 10µg of propidium iodide (PI)/ml, Hoechst 33342 (10µg/ml) and Hoechst 33358 (10µg/ml) were added to BMDMs at each distinct time-point post-infection (p.i.) with *E. coli* NCTC12900 and viability was analysed immediately. The number of PI-positive cells relative to the total number of nuclei per field was counted by automated fluorescence microscopy using LionHeart/FX Analyser. Each condition was assayed in triplicate and 4 fields were counted in each well. Cycloheximide (25µg/ml) was applied for 18hours as a positive control for cell death. Density of inocula were verified by plating.

### Quantitative Real-Time PCR (qPCR)

Total RNA was extracted from BMDMs using a PureLink RNA mini kit (Ambion) as per described in the manufacturer’s manual. Total RNA was reverse transcribed with a high-capacity cDNA archive kit (Applied Biosystems) and the cDNA was amplified using SYBR green-based real-time PCR (PowerUp SYBR green). Primer sequences are listed in **Table 1**. Relative quantification (RQ) of *Marcks*, *Rhob*, *Cdc42*, *Nos2*, *Fizz*, *Ym1*, *Arg1*, *Il-10* and *Mrc-1* mRNA levels were determined by the 2^-ΔΔCT^ method comparing genes of interest to endogenous control (*Rps13*).

**Table 1:** Primers used for SYBR Green qPCR.

Primer Pair	Forward	Reverse
*Marcks*	5'-CTCCTCCTTGTCGGCGGCCGG-3'	5'-GGCCACGTAAAAGTGAACGGC-3'
*Rhob*	5'-GACGGCAAGCAGGTGGAG-3'	5'-ATGGGCACATTGGGGCAG-3'
*Cdc42*	5'CGACCGCTAAGTTATCCACAG-3'	5'-AGGGCAGAGCACTCCACAT-3'
Nos2	5'-TTTGACAGAGCCACTGACATCCT-3'	5'-GAAAACTCATTGTCCCACATTGG-3'
*Flizz*	5'-ACTGCCTGTGCTTACTCGTTGACT-3'	5'-AAAGCTGGGTTCTCCACCTCTTCA-3'
*Ym1*	5'-TGTGGAGAAAGACATTCCAAG-3'	5'-AAGAGACTGAGACAGTTCAGG-3'
*Arg1*	5'-TTTTAGGGTTACGGCCGGTG-3'	5'-CCTCGAGGCTGTCCTTTTGA-3'
*1110*	5'-TTGAATTCCCTGGGTGAGAAG-3'	5'-TCCACTGCCTTGCTCTTATTT-3'
*Rps13*	5'-GGCCCACAAGCTCTTTCCTT-3'	5'-GACCTTCTTTTTCCCGCAGC-3'

### Immunoblotting

BMDM cell lysates were obtained following infection with *E. coli* NCTC12900 or from untreated samples. BMDMs were infected at MOI 20:1 for 15 min, and the media was then replaced with DMEM containing gentamicin (100 mg/ml) for a further 15 min. Samples were clarified, denatured with SDS loading buffer, and boiled for 5 minutes. A total of 20 mg protein lysate was fractionated on 12% SDS-PAGE, transferred to polyvinylidene fluoride membranes (Millipore) and probed with primary antibodies to murine MARCKS (1:2,000 dilution, ab51100, Abcam) (Lejonklou et al., 2012), CDC42 (1:500 dilution, 10155-1-AP, Proteintech), iNOS (1:500 dilution, 13120, Cell Signaling Technology) or β-actin (1:10,000 dilution, A3854, 3Sigma), incubated with horseradish peroxidase-conjugated secondary antibodies (Santa Cruz Biotechnology) and visualized using Pierce™ ECL HRP substrate (Thermofisher) and ImageQuant LAS 4000 scanner (GE Healthcare).

### NO Assay

To estimate NO release from BMDMs, Greiss reaction assay was carried out in accordance with the manufacturers protocol (Biotium). Optical density was read at 570 nm and the nitrate present in each sample was quantified using a standard curve, represented as NO_2_ µM.

### Enzyme Linked Immunosorbent Assay (ELISA)

Murine TNF-α, IL-6 and IL-10 production was detected in macrophage supernatants by ELISA according to the manufacturer’s protocol (Invitrogen/Thermofisher). Optical density was measured at 450 nm and cytokine concentrations were determined using a standard curve, expressed as pg/ml.

### Statistical Analysis

Numerical results are given as arithmetic means ± standard error of the means. Statistical differences were analysed using GraphPad Prism 6.0 statistical software [GraphPad Software Inc., San Diego, USA] by Student’s *t*-test or Two-way ANOVA. *P*-values of 0.05 or less [*p*<0.05] are considered statistically significant.

## Results

### Succinate Impairs and Itaconate Enhances Bacterial Phagocytosis by Macrophages

**Figure 1:**
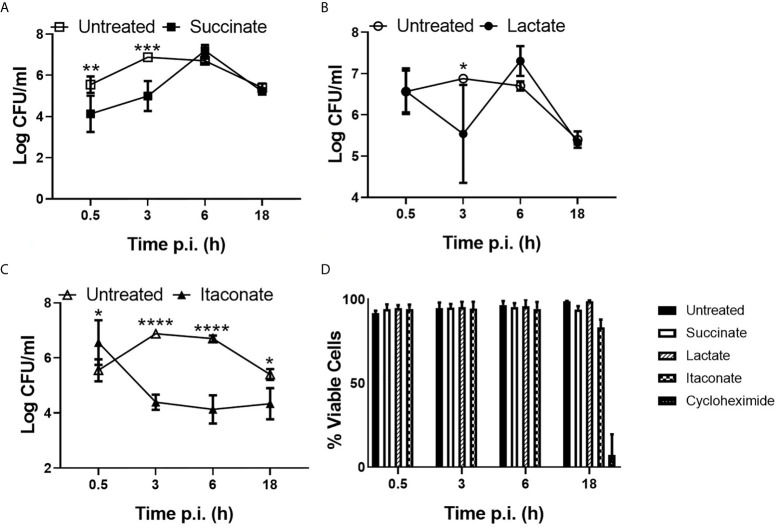
Phagocytic and killing capacity of macrophages are influenced by gut metabolites. BMDMs were pre-treated with **(A)** succinate (2.5 mM), **(B)** lactate (75 mM) or **(C)** itaconate (250 µM) for 3 hrs. BMDMs were infected with *E coli* NCTC12900 at MOI of 20:1. Bacterial burden is represented as Log CFU/ml. **(D)** Live/dead cell staining was carried out at distinct timepoints by addition of PI and Hoechst. Staining was visualized by fluorescent microscopy using Lionheart/FX. Mean values ± SEM are presented, graphs are representative of three independent experiments with three replicates each. Two way-Anova is shown, where significance is indicated as follows: p < 0.05 = *, p ≤ 0.01 = **, p ≤ 0.005 = ***, p ≤ 0.0001 = ****.

To gain insight into the potential impact of individual metabolites on macrophage function in response to infection, M0 BMDMs were pre-treated with succinate, lactate or itaconate followed by infection with *E. coli* NCTC12900 and the impact on bacterial phagocytosis and intracellular burden determined, as indicated by Log colony forming unit (CFU)/ml (**Figure 1**). BMDMs treated with succinate had a lower bacterial burden, as indicated by LogCFU/ml, at 30min and 3hr p.i. when compared to untreated BMDMs, indicating that succinate impairs engulfment of bacteria by macrophages. This initial difference was overcome later during the infection, with similar levels of intracellular bacteria recovered at 6hr and 18hr p.i. for both succinate-treated and untreated BMDMs (**Figure 1A**). This suggests that succinate impairs the ability of macrophages to control the intracellular growth of bacteria. Treatment of BMDMs with lactate did not appear to affect phagocytosis over the course of the infection (**Figure 1B**). Strikingly, itaconate-treated BMDMs had a significantly higher bacterial burden, as indicated by LogCFU/ml, 30min p.i. compared to untreated BMDMs and furthermore itaconate treatment significantly enhanced the ability of macrophages to control intracellular bacterial growth throughout the course of the infection, as indicated by LogCFU/ml (**Figure 1C**). All metabolites showed no effect on macrophage viability when compared to the positive control cyclohexamide (**Figure 1D**).

These results suggest that metabolites can have differing effects on macrophage function, with succinate impairing phagocytosis while itaconate significantly enhances phagocytosis and reduces the intracellular niche of *E. coli*.

### Succinate Impairs Bacterial Engulfment by Reducing Expression of RhoB, MARCKS and CDC42

**Figure 2:**
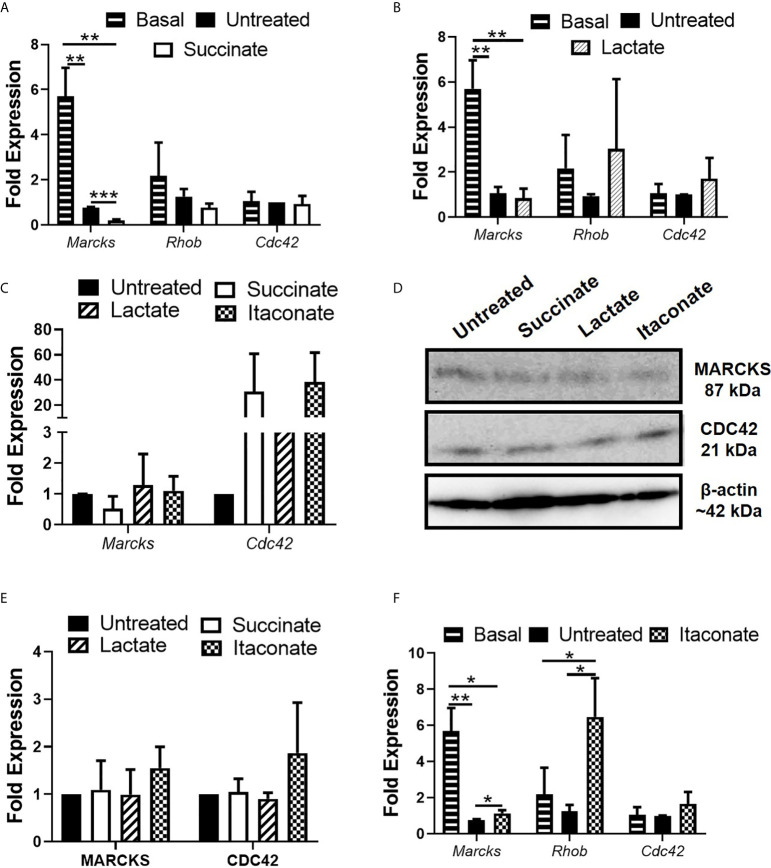
Impact of gut metabolites on pro-phagocytic marker expression. BMDMs were pre-treated with succinate (2.5 mM), lactate (75 mM) or itaconate (250 µM) for 3 hrs and infected *with E coli* NCTC12900 at MOI of 20:1. 15 minutes post infection, media was replaced with media containing 100µg/ml gentamicin and left for a further 15 minutes or for 2 hrs 45 min. **(A, B)** *Marcks*, *Rhob* and *Cdc42* gene expression was analyzed in succinate- and lactate-treated BMDMs 30 minutes p.i. with *E coli* NCTC12900. **(C)** *Marcks* and *Cdc42* gene expression was analyzed in metabolite-treated BMDMs 3hrs p.i. with *E* coli. **(D)** Protein analysis of MARCKS, RhoB and CDC42, followed by **(E)** densitometry were analyzed 3 hrs p.i. with *E* coli NCTC12900. **(F)** *Marcks*, *Rhob* and *Cdc42* gene expression was analyzed in itaconate-treated BMDMs 30 minutes p.i. with *E coli* NCTC12900. Mean values ± SEM are presented, graphs are representative of three independent experiments with three replicates each. Blots are representative of two independent experiments each, with one replicate each. **(G)** BMDMs were treated with each metabolite + LPS (100 µg/ml) for 24 hrs and RNA was taken to measure gene expression of Inos. Student *t*-test is shown, where significance is indicated as follows: p < 0.05 = *, p ≤ 0.01 = **, p ≤ 0.005 = ***.

To further investigate the impact of metabolites on phagocytosis, gene expression of pro-phagocytic regulators Ras homolog gene family member B (RhoB), cell division cycle-42 (CDC42) and myristoylated alanine-rich protein kinase C substrate (MARCKS) were assessed following metabolite pre-treatment and *E. coli* infection (**Figure 2**). MARCKS plays a role in actin reorganization, formation of the phagocytic cup and vesicle trafficking while RhoB and CDC42 are required for mannose receptor-mediated phagocytosis, a process whereby *E. coli* can be engulfed by macrophages (Zhang et al., 2005). In agreement with the previous observation that succinate impairs bacterial engulfment by macrophages, as indicated by LogCFU/ml (**Figure 1A**), succinate-treated BMDMs displayed significantly reduced mRNA expression of *Marcks*, *Rhob* and no difference in *Cdc42* 30 min after *E. coli* infection compared to untreated BMDMs (**Figure 2A**). The gene expression profile of *Marcks* was significantly higher in BMDMs not treated with any metabolite or infected with bacteria (basal) when compared to untreated or succinate-treated BMDMs p.i. with *E. coli* (**Figure 2A**). *Rhob* mRNA expression in basal BMDMs displayed an increasing trend when compared to untreated or succinate-treated BMDMs p.i. with *E. coli* while *Cdc42* remained indifferent (**Figure 2A**). These results suggest that succinate reduces the capacity of macrophages to engulf bacteria and furthermore to clear the infection. The gene expression profile of *Marcks* was significantly higher in basal BMDMs when compared to untreated or lactate-treated BMDMs p.i. with *E. coli* (**Figure 2B**). There was no difference in the mRNA expression of all target genes in lactate-treated BMDMs compared to untreated BMDMs following *E. coli* infection (**Figure 2B**). Expression of pro-phagocytic regulators was investigated at 3 hrs p.i. with *E. coli* in metabolite-treated BMDMs to check their expression at later stages of infection (**Figure 2C**). No real difference is noted in the gene expression profile of *Marcks* in succinate- and lactate-treated BMDMs (**Figure 2C**). The gene expression profile of *Cdc42* shows an increasing trend in succinate- and lactate-treated BMDMs 3 hrs p.i. with *E. coli* (**Figure 2C**). *Rhob* was unsuccessfully detected in succinate- and lactate-treated BMDMs 3 hrs p.i. with *E. coli*. Metabolite-induced changes to pro-phagoctyic genes noted at the 30 min timepoint prompted us to analyze the translation of this at a protein level at a later timepoint of 3 hrs, as a clear detection of protein 30 min p.i. was unsuccessful in previous attempts. Protein expression of MARCKS appeared to be reduced in succinate-treated BMDMs (**Figure 2D**). Protein expression of MARCKS remained indifferent following lactate-treatment (**Figure 2D**). Protein expression of RhoB was unsuccessful at this timepoint. Protein expression of CDC42 remained indifferent in succinate- and lactate-treated BMDMs (**Figure 2D**). However, normalization of the protein data against β-actin and quantification of immunofluorescence failed to show a significant difference (**Figure 2E**).

### Itaconate Enhances Phagocytosis by Increasing Expression of RhoB, MARCKS and CDC42

Itaconate-treated BMDMs displayed a significant increase in mRNA expression of *Marcks* and *Rhob* 30 min p.i. with *E. coli* compared to untreated BMDMs (**Figure 2F**). An increasing trend was noted in mRNA expression of *Cdc42* between untreated and itaconate-treated BMDMs 30 min p.i. with *E. coli* (**Figure 2F**). These observations are in agreement with the earlier observation of increased bacterial burden early during infection following treatment of macrophages with itaconate, as indicated by LogCFU/ml (**Figure 1C**). The gene expression profile of *Marcks* displayed an increased mRNA trend in basal BMDMs when compared to untreated or itaconate-treated BMDMs p.i. with *E. coli* (**Figure 2F**). At the 3 hr timepoint, itaconate-treated BMDMs had an increased trend in the mRNA expression of *Marcks* and *Cdc42* (**Figure 2E**). Protein expression of MARCKS remains indifferent and CDC42 appears to be increased 3 hrs p.i. with *E. coli* in itaconate-treated BMDMs (**Figure 2D**). However, normalization of the protein data against β-actin and quantification of immunofluorescence failed to show a significant difference (**Figure 2E**). These results demonstrate that itaconate enhances bacterial engulfment by macrophages through upregulation of pro-phagocytic regulators.

### Metabolites Influence NO Secretion From Macrophages in Response to *E. coli*

**Figure 3:**
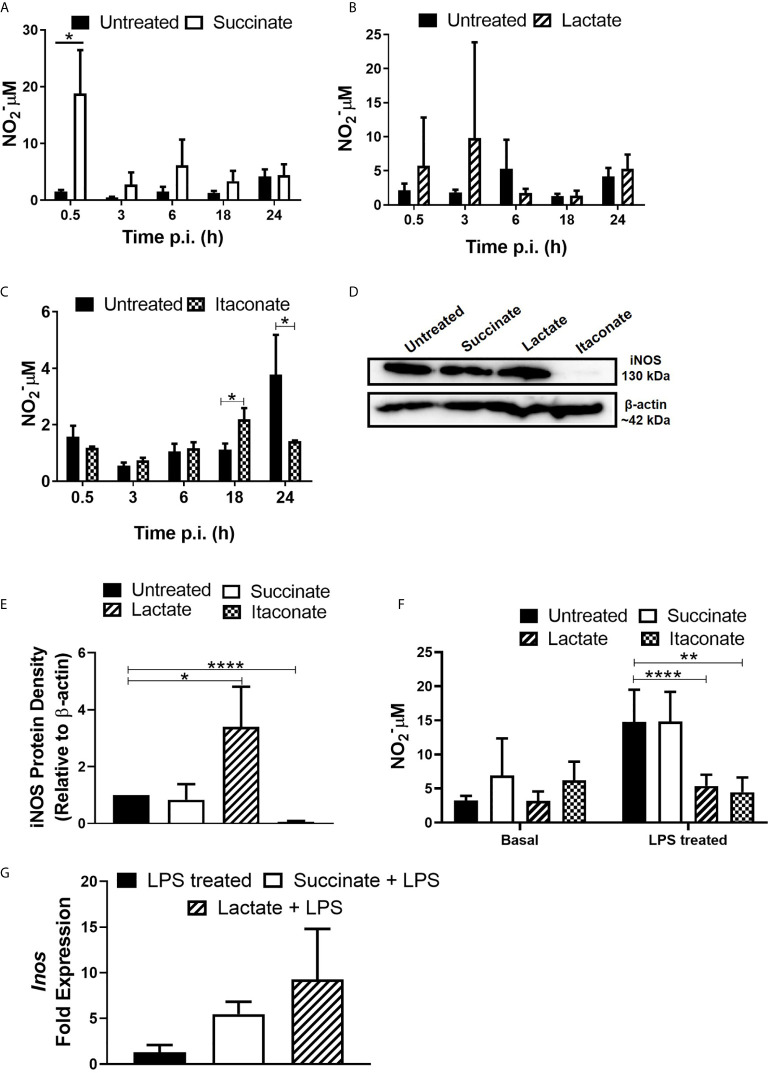
Gut metabolites boost macrophage NO production following E. coli infection and LPS exposure. BMDMs were pre-treated with succinate (2.5 mM), lactate (75 mM) or itaconate (250 µM) for 3 hrs. BMDMs were infected with *E. coli* NCTC12900 at MOI of 20:1. **(A–C)** Supernatants were taken at each timepoint and nitrate levels were measured by Greiss reaction. **(D, E)** Protein was isolated 24 hrs p.i. with *E. coli* NCTC12900 and iNOS was analysed, as confirmed by densitometry. **(F)** BMDMs were treated with each metabolite + LPS (100 µg/ml) for 24 hrs and supernatants were taken. **(G)** BMDMs were treated with each metabolite + LPS (100 µg/ml) for 24 hrs and RNA was taken to measure gene expression of *Inos*. Mean values ± SEM are presented, graphs are representative of three independent experiments with three replicates each. Blots are representative of three independent experiments each, with one replicate each. Student *t*-test is shown, where significance is indicated as follows: p < 0.05 = *, p ≤ 0.01 = ** and p ≤ 0.0001 = ****.

Next, the impact of metabolites on the bactericidal properties of macrophages was determined. Production of NO, a reactive intermediate that facilitates intracellular bacterial killing, was assessed in response to infection with *E. coli* (**Figure 3**). Treatment of BMDMs with succinate significantly increased NO secretion 30min p.i. with *E. coli* when compared to untreated BMDMs (**Figure 3A**), however there was no difference at later stages of infection. These observations appear to support the earlier observation that succinate-treated macrophages have a reduced bacterial burden initially with the number of intracellular bacteria increasing later in infection, as indicated by LogCFU/ml (**Figure 1A**). Lactate-treated BMDMs showed no difference in NO secretion across the distinct timepoints (**Figure 3B**). Itaconate-treated BMDMs showed a significant boost in NO secretion 18hr p.i. with *E. coli* compared to untreated BMDMs (**Figure 3C**). However, itaconate-treated BMDMs showed a significant reduction in NO secretion 24 hrs p.i. with *E. coli* (**Figure 3C**). A 24 hr timepoint was included to ensure detection of NO at a protein level. Protein expression of iNOS remained unchanged in succinate-treated BMDMs, enhanced in lactate-treated BMDMs and reduced in itaconate-treated BMDMs when compared to the untreated BMDMs p.i. with *E. coli* (**Figure 3D**). When we checked densitometry, succinate-treated BMDMs showed no difference in iNOS protein, while lactate-treated BMDMs had a significant enhancement in iNOS protein and itaconate-treated BMDMs showed significant reduction in iNOS protein (**Figure 3E**). Interestingly, exposure of BMDMs to each metabolite for 24 hrs did not influence basal NO secretion (**Figure 3F**). Addition of LPS was used to compare results obtained with *E. coli* infected BMDMs at 24 hrs to gain an insight of metabolite-induced changes to NO secretion in the context of inflammation. BMDMs were treated with each metabolite + LPS for 24 hrs, mimicking an inflamed environment (**Figure 3F**). Lactate- and itaconate-treated BMDMs showed a significant reduction in NO secretion at this time-point, while succinate-treated BMDMs remained indifferent when compared to the LPS only treated BMDMs (**Figure 3F**). *iNOS* facilitates the production of NO. After 24 hrs of metabolite + LPS, succinate- and lactate-treated BMDMs show no real difference in the gene expression of *iNOS* when compared to LPS control BMDMs (**Figure 3G**). The gene expression profile of *iNOS* in itaconate-treated BMDMs remained undetected. Taken together, these observations describe how metabolites induce NO secretion in response to infection whereby succinate boosts NO secretion, an observation affiliated with M1 pro-inflammatory macrophages, while lactate and itaconate reduce NO secretion, observations associated with anti-inflammatory M2 macrophages.

### Metabolites Alter Cytokine Secretion From Macrophages in Response to Infection

**Figure 4:**
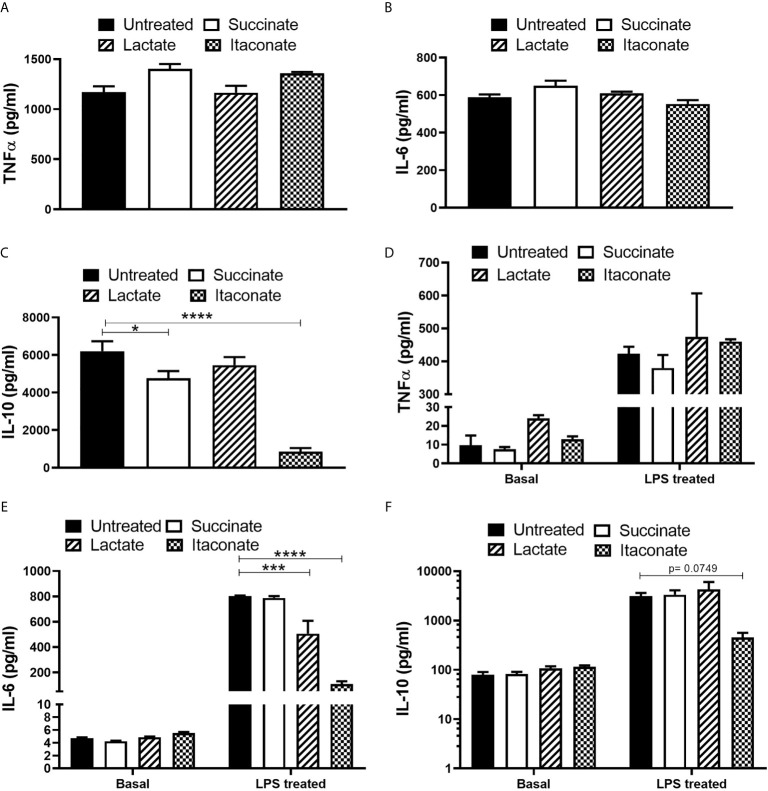
Cytokine secretion profile is altered following metabolite pre-treatment and *E. coli* infection or following metabolite-treatment -/+LPS exposure. BMDMs were pre-treated with succinate (2.5 mM), lactate (75 mM) or itaconate (250 μM) for 3 hrs and infected with *E. coli* NCTC12900 at an MOI of 20:1. **(A–C)** After 24 hrs, the concentrations of TNF-α, IL-6 and IL-10 in the supernatant were measured by ELISA. **(D–F)** BMDMs were treated with metabolites on their own for 24 hrs or with LPS for 24 hrs and cytokine secretion was analysed. Mean values ± SEM are presented, graphs are representative of three independent experiments with three replicates each. Student *t*-test is shown, where significance is indicated as follows: p < 0.05 = *, p ≤ 0.005 = *** and p ≤ 0.0001 = ****.

Further investigations into the impact of metabolites on bactericidal properties of macrophages was carried out (**Figure 4**). Succinate- and itaconate-treated BMDMs display increased secretion of pro-inflammatory TNF-α, while lactate-treated BMDMs showed no difference compared to untreated BMDMs 24 hrs p.i. with *E. coli* (**Figure 4A**). There was no difference in the secretion of pro-inflammatory IL-6 in response to each metabolite (**Figure 4B**). Succinate- and itaconate-treated BMDMs display decreased secretion of anti-inflammatory IL-10 in response to *E. coli* infection, compared to untreated BMDMs, while IL-10 secretion in lactate-treated BMDMs remain indifferent (**Figure 4C**). Interestingly, exposure of BMDMs to each metabolite for 24 hrs did not influence basal pro- and anti-inflammatory cytokine secretion (**Figures 4D** **–F**). In response to the inflammatory stimulus LPS, metabolite-treated BMDMs display no real difference in TNF-α (**Figure 4D**). Lactate- and itaconate-treated BMDMs display a significant decrease in IL-6 (**Figure 4E**). Succinate- and lactate-treated BMDMs display no difference in IL-10 secretion, while itaconate-treated BMDMs show a reduced trend in IL-10, however, this is not significant (**Figure 4F**). Thus, we note the secretion of both pro- and anti-inflammatory cytokines from metabolite-treated BMDMs at a physiological level as well as in response to infection and inflammation.

### Metabolites Alter the Expression of Macrophage Phenotypic Markers

**Figure 5:**
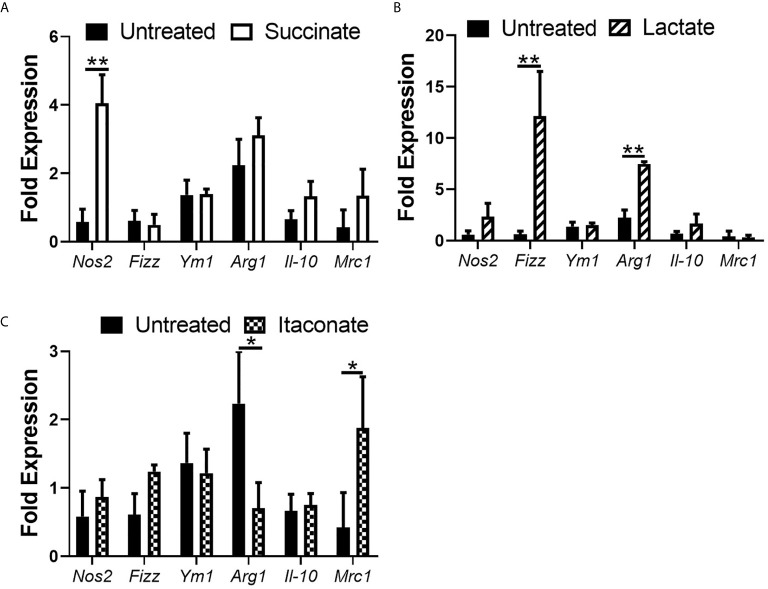
Impact of metabolite treatment on macrophage phenotypic marker expression. BMDMs were treated with **(A)** succinate (2.5 mM), **(B)** lactate (75 mM) or **(C)** itaconate (250 μM) for 3 hrs followed by RNA isolation. Gene expression of *Inos*, *Fizz*, *Ym1*, *Arg1*, *Il-10* and *Mrc-1* were analyzed. Mean values ± SEM are presented, graphs are representative of three independent experiments with three replicates each. Student *t*-test is shown, where significance is indicated as follows: p < 0.05 = *, p ≤ 0.01 = **.

Up until now, no real difference in NO and pro- and anti-inflammatory cytokine secretion has been noted in metabolite-treated BMDMs without introduction of infection or inflammatory stimuli. We next investigated the impact of each individual metabolite on macrophage genetic phenotype by assessing mRNA expression of M1/M2 markers in M0 BMDMs (**Figure 5**). mRNA expression of the M1 pro-inflammatory gene marker *Nos2* was significantly increased in succinate-treated BMDMs compared to untreated BMDMs (**Figure 5A**). There was no statistically significant difference in mRNA expression of the M2 anti-inflammatory markers *Fizz*, *Ym1*, *Arg1*, *Il-10* and *Mrc1* in succinate-treated BMDMs infection compared to untreated BMDMs (**Figure 5A**). These results agree with current literature which shows that succinate promotes a pro-inflammatory M1-phenotype (Jha et al., 2015). Lactate-treated BMDMs show a significant increase in mRNA expression of *Fizz* and *Arg1* when compared to untreated BMDMs (**Figure 5B**). Although not statistically significant, lactate-treatment increases the mRNA expression of both *Nos2* and *Il-10* in BMDMs, while *Mrc1* remains indifferent (**Figure 5B**). Itaconate-treated BMDMs show no difference in mRNA expression of *Nos2*, *Fizz*, *Ym1* and *Il-10* (**Figure 5C**), however, mRNA expression of *Mrc1* is significantly increased and *Arg1* is significantly decreased compared to untreated BMDMs (**Figure 5C**). These results describe the basal effects of gut-associated metabolites on macrophage genetic marker phenotypes.

## Discussion

The intestinal lumen is a rich metabolic environment. The diverse population of the gut microbiota converts ingested food or host products into metabolites, which target either the intestinal microbial population or host cells. Another newly understood source of gut metabolites is primed immune cells, particularly macrophages. Within the gut, the largest pool of macrophage reside, forming a crucial part of our body’s defense (Lee et al., 1985). Macrophage recognize, engulf and digest invading pathogens, a process termed phagocytosis, being fundamental to the control of infection (Rougerie et al., 2013). The secretion of three metabolites succinate, lactate and itaconate is an outcome of macrophage activation, however, how these metabolites influence biological functioning of resident macrophages particularly in response to bacterial infection remains poorly understood (Jha et al., 2015).

In this work, we have demonstrated how succinate limits the ability of macrophage to engulf *E. coli* while itaconate boosts phagocytic capability, thereby reducing the intracellular niche of this pathogen and promoting clearance of the infection, with lactate showing no difference in macrophage functioning. *E. coli* NCTC12900 was chosen due to pathogenic *E. coli* dominantly infecting patients with inflammatory bowel disease (IBD) (Walker et al., 2011). Treatment of BMDMs with succinate impaired macrophage ability to engulf *E. coli* 30 min p.i. Succinate has been previously associated with M1, pro-inflammatory macrophages that are less phagocytic (Leidi et al., 2009; Tannahill et al., 2013). Interestingly, BMDMs treated with succinate and infected with non-pathogenic *E. coli* K-12 MG1665 displayed a similar outcome, whereby engulfment rates of BMDMs were reduced, as indicated by reduced CFUs 30 minutes p.i. (see **Supplementary Figure 2A**). While lactate secretion is associated with rapidly proliferating M1, pro-inflammatory macrophages, the literature also describes how lactate induces M2-like macrophage, that are known to efficiently phagocytose (Leidi et al., 2009; Colegio et al., 2014; Chen et al., 2017, Zhang et al., 2020). In the current study, lactate did not influence macrophage rate of engulfment following infection with *E. coli*. However, we see the opposite effect when infected with non-pathogenic *E. coli* K-12 MG1665, whereby lactate treated BMDMs have reduced engulfment rates compared to untreated BMDMs, as indicated by reduced CFUs (see **Supplementary Figure 2B**). There is limited literature describing the outcome of itaconate-treated macrophages in response to infection. Interestingly, we show that BMDMs treated with itaconate display stronger phagocytic capabilities compared to untreated BMDMs, with intracellular killing abilities improved as bacterial burden is overall reduced. Whereas, upon infection with non-pathogenic *E. coli* K-12 MG1665, no real difference in engulfment rates or killing abilities are noted (see S**upplementary Figure 2C**). Previous studies have focused on intracellular itaconate whereby immune-responsive gene 1 (IRG1)-deficient immune responses *in vivo* succumbed rapidly to *Mycobacterium tuberculosis* infection (Nair et al., 2018). IRG1 is responsible for itaconate production in activated macrophages. However, similar-scale defects *in vitro* using IRG1-deficient macrophages were not reported. These studies focus on intracellular itaconate rather than endogenous, externally available itaconate and how this impacts macrophage function and pathogenesis of infection. Our work illustrates a dual role of itaconate on macrophage biological functioning, whereby itaconate treated macrophages limits *E. coli* infection intracellularly, distinctive of an M1 macrophage, however, the rate of engulfment is enhanced in these macrophages, characteristic of an M2 macrophage. Indeed, similar outcomes of engulfment and intracellular killing of these metabolite-treated macrophages are noted when comparing infection of pathogenic *E. coli* NCTC12900 to non-pathogenic *E. coli* K-12 MG1665 (see **Supplementary Figure 2**). BMDMs were used as a model cell type to mimic macrophages derived from the intestine. Interestingly, mucosal macrophages have a high level of turnover from BM, hence the use of BMDMs in our studies (De Schepper et al., 2018). Further work using colonic lamina propia macrophages and the move to an *in vivo* murine model of infection would clarify preliminary data shown using BMDMs.

Following on from the observed impact on bacterial phagocytosis, we questioned whether succinate, lactate and itaconate may influence genes involved in the initial engulfment and uptake of bacteria by macrophages 30min p.i. Key mediators of actin rearrangements and vesicle trafficking are the RhoGTPases RhoB and CDC42, which have been shown to be important for the initial formation of the phagocytic cup, engulfing invading pathogen (Rougerie et al., 2013). Both RhoB and CDC42 are required for mannose receptor-mediated phagocytosis of pathogens (Zhang et al., 2005). Felipe et al. confirm that mannose receptors mediate the phagocytosis of *E. coli* (Felipe et al., 1991 *).* Thus, we investigated the impact of metabolites on these particular engulfment mediators in an effort to uncover a mechanism underlying the observed effects on phagocytosis. Macrophages have been shown to express high levels of MARCKS, an actin cross-linking protein (Carballo et al., 1999). In particular, this increased MARCKS expression is found in areas of the cell where actin filaments associate with the plasma membrane, and its expression is associated with regulation of cell motility (Myat et al., 1997; Carballo et al., 1999). This could explain why we see high basal expression of MARCKS in untreated and uninfected (basal) macrophages. We observed a significant reduction in mRNA of *Marcks* and a reduced gene expression profile of *RhoB* in succinate-treated BMDMs compared to untreated BMDMs, reinforcing the diminished capacity to engulf *E. coli*. Zhang et al . reported low succinate levels in efferocytes (macrophages that engulf dying cells), agreeing with our observation that addition of succinate in macrophage microenvironment ablates phagocytic ability (Zhang et al., 2019). Moreover, succinates association with M1, pro-inflammatory macrophages have diminished phagocytic ability (Leidi et al., 2009; Jha et al., 2015). At 3 hrs p.i., gene expression of *Marcks* shows a reduced trend in succinate-treated BMDMs, while *Cdc42* shows an increased trend. Perhaps CDC42 is partaking in the transportation of bacteria-containing vesicles to aid eradicating infection at the timepoint. A recent study showed how lactate acts upon GPR81 inducing alternatively activated M2 macrophage in response to LPS, which in turn reduces the production of pro-inflammatory factors, weakens the phagocytosis of macrophages, and inhibits the immune response (Wang et al., 2020). Our results agree with this whereby macrophage pro-phagocytic regulators show significant reduction or no real difference in mRNA expression following lactate treatment in response to pathogenic bacteria. At 3 hrs p.i., gene expression of *Marcks* displayed no difference in lactate-treated BMDMs, while *Cdc42* displayed an increased trend. Interestingly, itaconate-treated BMDMs displayed a significant increase in mRNA of *Marcks* and an increase in mRNA of *Rhob*, agreeing with initial phagocytic observations. At 3 hrs p.i., protein levels of MARCKS and CDC42 show an increased trend. These observations are novel as currently there is no published data outlining the impact of itaconate on macrophage engulfment regulators. Thus, these cells can effectively act upon intracellular bacterial levels, internally exterminating the engulfed pathogen. We were unable to detect RhoB gene and protein levels at the 3 hr timepoint, suggesting that future work may need to assess expression of this factor earlier during infection.

Primed macrophages release NO, which is an important antimicrobial mechanism accompanying engulfment to counteract microbial infection (Bogdan, 2001). To determine whether the reduced bacterial load noted in succinate treated macrophages early in response to infection with *E. coli* is due to an inability of macrophages to engulf bacterial cells or due to increased production of antimicrobial mediators, we analyzed the production of NO in untreated and succinate-treated BMDMs following *E.coli* infection. We observed a significant boost in NO production in macrophages treated with succinate, characteristic of M1, pro-inflammatory macrophage. Indeed, central to the pro-inflammatory switch is the expression of iNOS, which generates large quantities of NO. Bailey et al. reveal how NO modulates succinate levels and in the context of infection, could have paracrine effects impacting engulfment and eradication of potential pathogens (Bailey et al., 2019). However, there is a lack of literature outlining the impact succinate has on NO production. We show how succinate treated macrophages maintain NO production 24 hrs p.i. with *E. coli* and in response to the inflammatory stimulus LPS. However, the level of production is not sufficient to significantly eradicate the intracellular bacterial niche. Surprisingly, lactate-treated BMDMs show an increase in NO production during infection with *E. coli* and decrease in NO production in response to the inflammatory stimulus LPS. This observation is contradicting, however, agrees with literature describing how lactate induces the expression of genes affiliated with M2 macrophages (Ohashi et al., 2013), where NO is not a characteristic secretory product of these cells. Furthermore, Miles et al. show no correlation between endogenous NO production and lactate content of alveolar macrophages, describing how NO does not directly affect cellular energy metabolism (Miles et al., 1998). In the context of inflammation, we observe a significant decrease in NO production in macrophages treated with lactate + LPS, supporting the knowledge that lactate is associated with inducing M2 macrophages. Itaconate-treated BMDMs show a significant boost in NO production 18 hrs p.i. and a significant reduction in NO production 24 hr p.i. This finding is complemented by exposing macrophages to itaconate + LPS for 24 hrs, whereby gene expression of *iNOS* was undetected supporting the reduced NO production in these macrophages. Of recent, the activation of nuclear factor erythroid 2-related factor 2 (NRF2) in response to LPS was shown to be ablated in IRG1-deficient macrophages (Bambouskova et al., 2018), pinpointing itaconate as an activator of NRF2 (data we have confirmed but not shown). NRF2 is a well-studied transcription factor induced in response to oxidative stress, protecting cells against cytotoxic effects. NO secretion correlates with oxidative stress, and so perhaps the limited secretion 18 hrs p.i. and post itaconate treatment displays how the induction of NRF2 has worn off, enabling significant yet low levels of NO production, aiding in eradicating bacterial infection. Thus, perhaps itaconate treatment initially induces characteristics of an M2 macrophage, however, enables a switch to characteristics of an M1 macrophage in response to long-term pathogenic infection. Surprisingly, itaconate-treated BMDMs have reduced NO secretion 24 hours p.i. with *E. coli*. Perhaps if we investigated CFUs at this timepoint, we would see bacterial numbers recovering to that of the control group or increasing, describing how the pathogen is becoming successful again. Further work is required to fully support this. Interestingly, the influence all three metabolites have on NO production in response to non-pathogenic *E. coli* MG1665 does not replicate results obtained in response to pathogenic *E. coli* NCTC12900 (**Supplementary Figures 2D–F**). Indeed, *E. coli* possess NO-detoxifying enzymes that accounts for successful *E. coli* infection in macrophages (Stevanin et al., 2007; Baptista et al., 2012). Interestingly, the contribution of these NO-detoxifying enzymes exhibit a time-dependent profile, whereby bacterial protection only occurred in macrophages for time-periods longer than 8 hrs (Baptista et al., 2012). Perhaps this could explain why bacterial counts initially drop in succinate-treated macrophages, due to increase NO production, and recover at later-timepoints p.i. with *E. coli*.

Next the impact of metabolites on the cytokine response of macrophages to infection and inflammation was determined. We observed increasing trends in TNF-α and IL-6, as well as a significant decrease in IL-10 secretion in macrophages treated with succinate, agreeing with current literature to date affiliating succinate with M1, pro-inflammatory macrophages (Jha et al., 2015). There was no difference in TNF-α, IL-6 and IL-10 in lactate-treated macrophages. Interestingly, macrophages treated with lactate + LPS had reduced IL-6, agreeing with literature outlining how lactate exerts suppressive effects on LPS stimulated pro-inflammatory cytokine production in macrophages. It is clear lactate influences cytokine secretion in the context of inflammation. BMDMs treated with itaconate displayed increased secretion of TNF-α and a significant reduction in IL-10 secretion compared to untreated BMDMs in response to infection. This could describe why we see a reduction in NO secretion 24 hrs p.i. with *E. coli*, perhaps describing how CFUs may recover or increase at later stages of infection. Contradicting roles for itaconate have been described whereby this metabolite can induce both pro-inflammatory and anti-inflammatory cytokines; itaconate inhibits succinate dehydrogenase (SDH), enhancing succinate levels which induces the transcription factor HIF-1α, causing the secretion of IL-1β (Tannahill et al., 2013). However, NRF2, the transcription factor that itaconate induces in macrophages, inhibits the production of IL-1β and IL-6 by directly binding to their promoters (Kobayashi et al., 2016), an observation we replicate with IL-6 in itaconate + LPS treated macrophages. In contrast, most recent evidence have described how itaconate treated BMDMs suppressed IL-1β secretion but not pro-IL-1β levels, and, surprisingly, strongly enhanced LPS-induced interferon-β secretion (Swain et al., 2020). Furthermore, macrophages treated with an itaconate derivative 4-octyl-itaconate decreased LPS-induced IL-10 protein levels, data that we show (although not significant, *p* = 0.0749), which is likely to be a consequence of reactive oxygen species (ROS) detoxification after NRF2 induction (Mills et al., 2018).

It remains poorly understood if these gut-associated metabolites can directly influence macrophage polarization considering macrophage are continuously surrounded by them, particularly in an inflamed intestine. The pro-inflammatory marker *Nos2* is significantly boosted in succinate-treated BMDMs. This observation would agree with current literature to date that associate’s succinate with a pro-inflammatory macrophage state (Mills et al., 2016). This observation supports earlier results whereby succinate-treated BMDMs infected with *E. coli* secreted large volumes of NO during early stages of infection. Therefore, succinate inducing *Nos2* expression may account for its secretion. BMDMs treated with lactate had significantly enhanced mRNA expression of the anti-inflammatory, M2 markers *Fizz* and *Arg1*. One study showed that lactic acid stabilizes HIF-1α which is required for the induction of ARG1 (Colegio et al., 2014). No real difference is observed in phenotypic marker expression in macrophages treated with itaconate, other than a significant increase in mRNA of the M2 marker *Mrc1* and a significant decrease in mRNA of the M2 marker *Arg1*. Interestingly, MRC-1 has been shown to play an important role in phagocytosis of *E. coli*, agreeing with earlier observations, and supporting our acknowledgement of mannose receptor-mediated phagocytosis of *E. coli* in metabolite-treated cells (Hashino et al., 2012). Defining macrophage phenotype following itaconate treatment has been proved more complex as two studies have indicated that prevention of the differentiation of M2 macrophages increased IRG1 expression and itaconate production. Peroxisome proliferator-activated receptor-γ (PPAR-γ) is a master regulator of fat cell function and adipogenesis, has been shown to be associated with M2 macrophage activation. PPAR-γ deficiency led to an enhanced expression of IRG1 (Nelson et al., 2018). Furthermore, a decrease in IRG1 and itaconate caused by micro-RNA (miR)-093, was required to allow for M2 macrophage polarization (Ganta et al., 2017). Indeed, the broad spectrum of macrophage functions depends on both heterogeneity and plasticity of these cells, which are highly specialized in sensing the microenvironment thus modifies their properties accordingly. Defining an exact metabolite-induced phenotype is not as simple or clear cut and further work is required to fully understand the phenotypic fate of macrophages following exposure to intestinal metabolites as gene expression profiling does not account for differences in functional phenotype and cannot reveal the complexity of biological samples (Kosmides et al., 2013).

In sum, our studies indicate that gut-associated metabolites succinate and itaconate modulate host responses to *E. coli* infection, whereby phagocytosis and bactericidal activities are altered, thereby reducing the intracellular niche of this pathogen and promoting clearance of infection. This work delineates new insights into the interplay between gut-associated metabolites and macrophage biological functioning.

## Data Availability Statement

The raw data supporting the conclusions of this article will be made available by the authors, without undue reservation.

## Ethics Statement

The animal study was reviewed and approved by Animal Research Ethics Committee (AREC), Trinity College Dublin and the Irish Health Products Regulatory Authority.

## Author Contributions

SC conceived ideas and oversaw the research programme. AO’C designed and performed the experiments. ED, NI, SS, and KM assisted with experiments. SC and AO’C analyzed data and wrote the manuscript. All authors contributed to the article and approved the submitted version.

## Funding

This research is supported by a Starting Investigator Research Grant from Science Foundation Ireland [SFI] [grant number 11/SIRG/B2099] and a Litwin IBD Pioneer award [Award number 47894] from the Crohns and Colitis Foundation of America, both awarded to SCC. NI is supported by European Union’s Horizon 2020, Marie Sklodowska-Curie COFUND (grant number 754535).

## Conflict of Interest

The authors declare that the research was conducted in the absence of any commercial or financial relationships that could be construed as a potential conflict of interest.

## Supplementary Material

Supplementary Figure 1 Metabolite dose-dependent assay. BMDMs were pre-treated with various concentrations of **(A)** succinate, **(B)** lactate or **(C)** itaconate for 3 hrs. BMDMs were infected with *E. coli* NCTC12900 at MOI of 20:1. Bacterial burden is represented as Log CFU/ml. Mean values ± SEM are presented, graphs are representative of three independent experiments with two replicates each. Student *t*-test is shown, where no statistical significance is observed.

Supplementary Figure 2 Impact of metabolite treatment on phagocytic and killing abilities of non-pathogenic *E. coli* MG1665. BMDMs were pre-treated with succinate (2.5 mM), lactate (75 mM) or itaconate (250 µM) for 3 hrs. BMDMs were infected with *E. coli* MG1665 at MOI of 20:1. **(A–C)** Bacterial burden is represented as Log CFU/ml. **(D–F)** Supernatants were taken at each timepoint and nitrate levels were measured by Greiss reaction. Mean values ± SEM are presented, graphs are representative of three independent experiments with three replicates each. Two way-Anova and Student *t*-test are shown, where significance is indicated as follows: p < 0.05 = *, p ≤ 0.01 = **, p ≤ 0.005 = ***.
