# Supplementary figures and images for: Intestinal Metabolites Influence Macrophage Phagocytosis and Clearance of Bacterial Infection
Source: Front Cell Infect Microbiol. 2021 Jul 19;11:622491. doi: 10.3389/fcimb.2021.622491 (PMC8327167; doi:10.3389/fcimb.2021.622491)

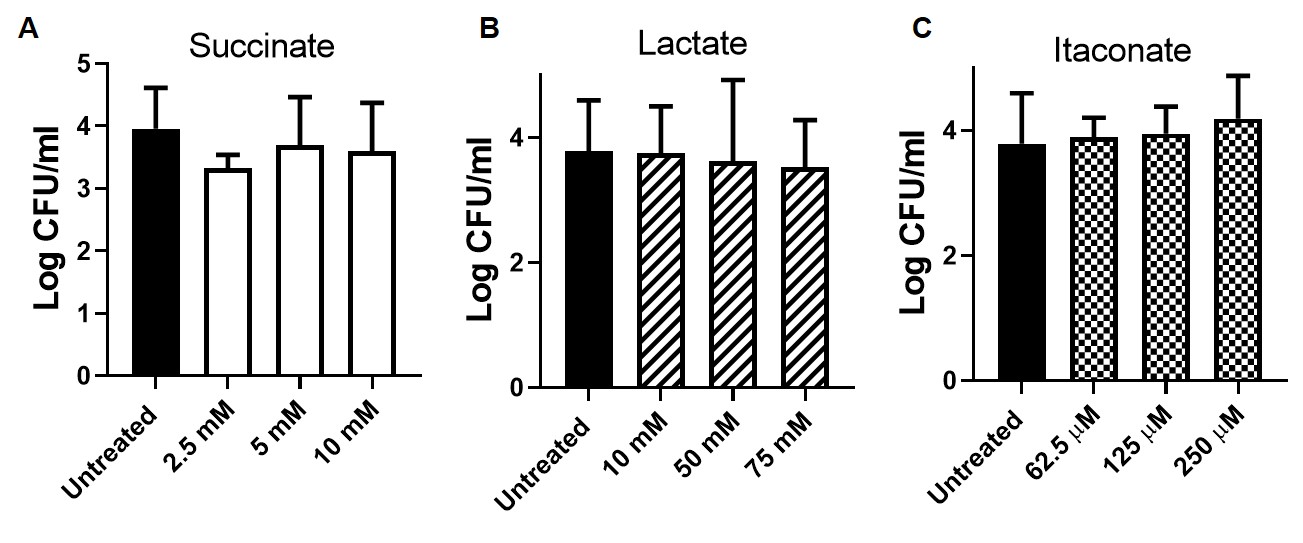

Supplement: Supplementary Figure 1 — Metabolite dose-dependent assay. BMDMs were pre-treated with various concentrations of (A) succinate, (B) lactate or (C) itaconate for 3 hrs. BMDMs were infected with E. coli NCTC12900 at MOI of 20:1. Bacterial burden is represented as Log CFU/ml. Mean values ± SEM are presented, graphs are representative of three independent experiments with two replicates each. Student t-test is shown, where no statistical significance is observed. [file Image_1.jpeg]

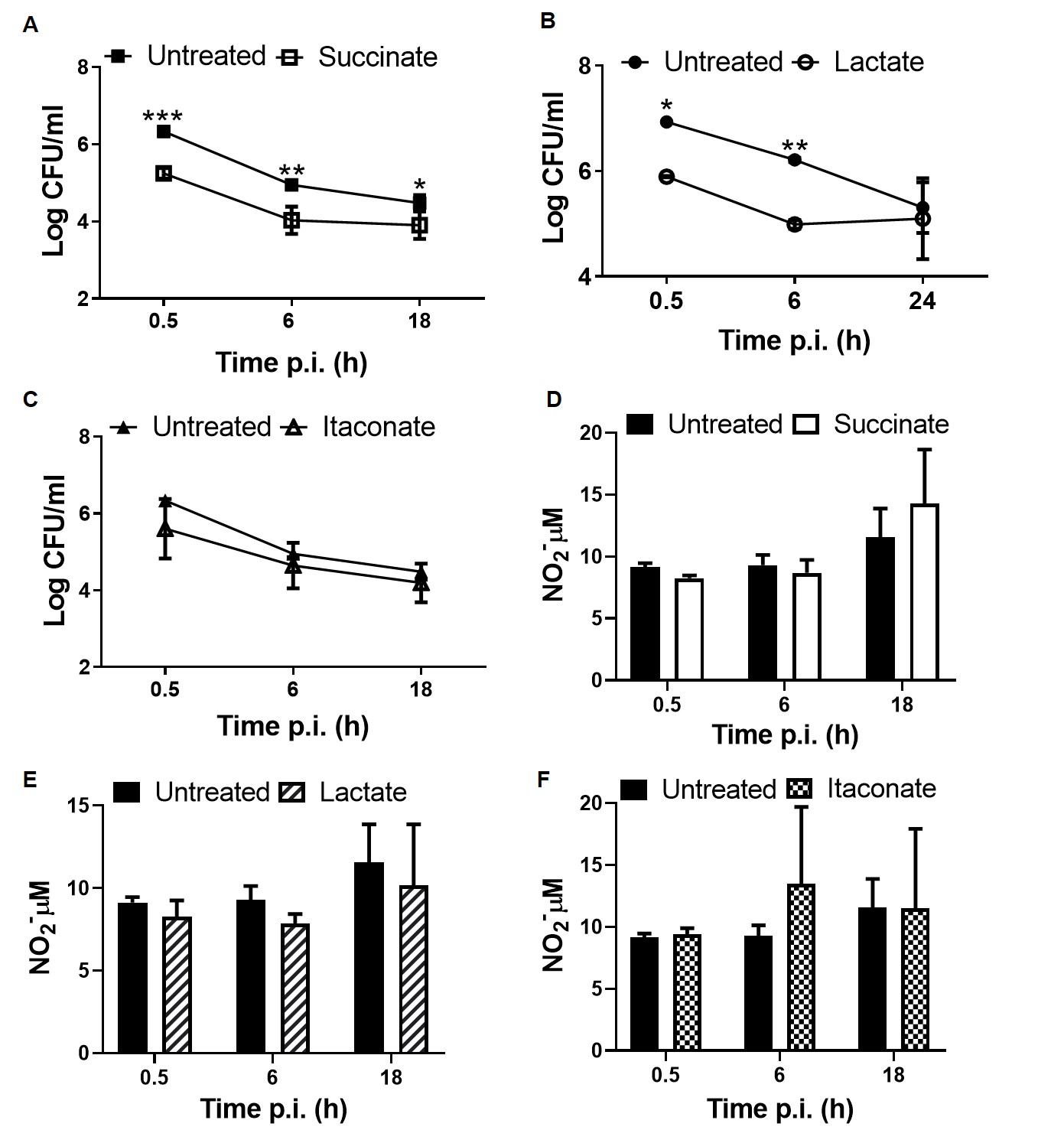

Supplement: Supplementary Figure 2 — Impact of metabolite treatment on phagocytic and killing abilities of non-pathogenic E. coli MG1665. BMDMs were pre-treated with succinate (2.5 mM), lactate (75 mM) or itaconate (250 µM) for 3 hrs. BMDMs were infected with E. coli MG1665 at MOI of 20:1. (A–C) Bacterial burden is represented as Log CFU/ml. (D–F) Supernatants were taken at each timepoint and nitrate levels were measured by Greiss reaction. Mean values ± SEM are presented, graphs are representative of three independent experiments with three replicates each. Two way-Anova and Student t-test are shown, where significance is indicated as follows: p < 0.05 = *, p ≤ 0.01 = **, p ≤ 0.005 = ***. [file Image_2.jpeg]
